# Influence of Cusp Coverage Design and Hybrid Resin–Ceramic Materials on the Biomechanical Performance of Partial Coverage Restorations

**DOI:** 10.3390/jfb16110394

**Published:** 2025-10-22

**Authors:** Abdullah Alshehri

**Affiliations:** Conservative Dental Science Department, College of Dentistry, Prince Sattam Bin Abdulaziz University, Alkharj 11942, Saudi Arabia; am.alshehri@psau.edu.sa

**Keywords:** cusp coverage, hybrid ceramics, partial coverage, fracture, marginal adaptation

## Abstract

Restoration of structurally compromised teeth often requires cusp coverage, yet the influence of preparation design and material type on performance remains unclear. This study evaluated the effect of cusp coverage design and hybrid resin–ceramic material on the marginal adaptation and fracture resistance of partial coverage restorations in mandibular molars. Eighty extracted teeth were prepared for indirect restorations and allocated to four groups (*n* = 20) according to design, either functional cusp coverage (FC) or complete cusp coverage (CC) and material, either GC Cerasmart (CS) or VITA Enamic (EN). Restorations were bonded with dual-cure resin cement, thermocycled, and subjected to cyclic loading. Fracture load, marginal adaptation, and failure mode were evaluated (α = 0.05). CC-CS and CC-EN exhibited significantly higher fracture loads than FC-CS and FC-EN (*p* < 0.001), while no difference was found between materials within each design. For marginal adaptation, CS showed significantly greater marginal gaps than EN in both designs (*p* < 0.001). CC designs demonstrated a higher proportion of repairable failures (Type I and II), whereas EN showed more catastrophic fractures. Within the limitations of this in vitro study, cusp coverage design significantly affected fracture resistance, while material type primarily influenced marginal adaptation. Both hybrid resin–ceramics provided acceptable mechanical performance for partial coverage restorations.

## 1. Introduction

Restoring posterior teeth with extensive structural loss remains a clinical challenge [1,2,3]. Conservative methods are frequently hindered by the necessity to eliminate healthy tooth structure for full coverage restorations. While preserving healthy tooth structure is crucial for improving clinical outcomes and sustaining long-term biomechanical performance, as evidenced by numerous research [1,2,3], the clinical deployment of appropriate pretreatment strategies is essential to strengthen compromised teeth [4,5,6,7,8]. Partial coverage restorations such as onlays, overlays and endocrowns has emerged as a conservative alternative, facilitated by developments in adhesive dentistry [4]. These restorations demonstrate clinical efficacy akin to that of full-coverage crowns while conserving a greater amount of natural tooth structure. Furthermore, current advancements in adhesive systems and restorative materials have reinforced the principles of biomimetic preparation [9,10,11,12]. Historically, CC and overlay reductions have been employed to reinforce compromised tooth structure, especially in severely damaged teeth [4,13,14]. Recently, preparation designs have progressed to defect-oriented, morphology-based methodologies [15]. Computer-aided design and manufacturing (CAD-CAM) systems have significantly enhanced the precision and reproducibility of these restorations, achieving adhesive gaps of less than 50 µm [4,16,17,18,19]. Concurrently, hybrid ceramic materials have garnered heightened popularity owing to their advantageous mechanical, esthetic, and clinical handling characteristics [16,18,19,20,21,22,23]. According to manufacturers, hybrid ceramics provide advantages including quick milling, reduced hardness, elevated flexural strength, ease of polishing, and enhanced elasticity for stress absorption [24,25,26].

Hybrid ceramics can be broadly classified into two types according to their microstructure: polymer-infiltrated ceramic networks (PICNs) and dispersed fillers (DFs) [23]. VITA Enamic (EN) (VITA Zahnfabrik, Bad Säckingen, Germany) is a representative PICN, composed of 86% feldspathic ceramic network and 14% polymer by weight, offering a flexural strength of 150–160 MPa [24,27]. EN composition comprises 86% feldspathic ceramic and 14% polymer by weight, resulting in a flexural strength of around 150–160 MPa. This micro-structure merges the rigidity of ceramics with the flexibility of polymers, with the goal of emulating the mechanical properties of dentin. Since its launch in 2013, EN has found applications in various areas such as inlays, onlays, partial crowns, and implant-supported restorations [28,29,30].

On the other hand, GC Cerasmart (CS) (GC Corp., Tokyo, Japan), a nanoceramic derived from DFs, demonstrates a superior flexural strength of around 238 MPa, making it appropriate for use in both anterior and posterior restorations [31,32,33]. The flexibility of the resin matrix is suggested to absorb functional stress and mitigate fracture risk. Numerous aspects influence the performance of partial coverage restorations, encompassing cavity dimensions [5,7], material characteristics [20,34], and the adhesive agent employed [32]. Importantly, the degree of cusp coverage whether it be functional or complete, has been demonstrated to have a significant impact on biomechanical performance [5,7]. Factors unique to each patient, including the quality of teeth, the distribution of occlusal stress, and habits related to oral hygiene, play a significant role in determining long-term clinical success [4,5,6,7,8].

FC may more effectively preserve tooth structure, whereas CC can provide superior reinforcement. However, this decision influences the probability of catastrophic vs. repairable failure modes, highlighting the necessity for direct comparison [20,21,23]. Despite the widespread clinical use of both hybrid ceramic materials and these preparation designs, limited research exists on how their interaction influences fracture resistance and marginal adaptation under simulated oral conditions. Therefore, the present study aimed to evaluate the biomechanical performance of partial coverage restorations fabricated from two hybrid ceramics—EN and CS—using two preparation designs: FC and CC, following thermomechanical aging. Consequently, two null hypotheses were formulated: (1) The design of cusp coverage does not influence fracture resistance or marginal adaption. (2) The specific hybrid ceramic composition does not influence fracture resistance or marginal adaption.

## 2. Materials and Methods

### 2.1. Sample Size Calculation

Based on a previous study of Hofsteenge et al., sample size was calculated [5]. The smallest sample size was decided to be 15 samples per group, with 80% power and 5% significance. This was adjusted to *n* = 20 to account for approximately 25% dropouts, resulting in a total of 80 as the sample size.

### 2.2. Specimens Preparation

This study was approved by the Standing Committee of Bioethics Research of Prince Sattam bin Abdulaziz University, under approval number SCBR-325/2024. Eighty intact human mandibular molars were obtained from patients aged 20 to 70 years. Teeth were extracted for orthodontic and periodontal purposes. Each selected molar had almost identical dimensions and shape to reduce anatomical heterogeneity. The measurement of the tooth was conducted mesiodistally and buccolingually at the cementoenamel junction with a digital caliper (±0.01 mm precision; 784EC; Sona Enterprises, Los Angeles, CA, USA). Teeth deviating more than ±10% from the average crown measurements were eliminated. Only teeth devoid of cavities, fractures, restorations, abrasion, or developmental anomalies were included. Those exhibiting evident craze lines or having undergone endodontic treatment were excluded. Specimens were scaled with an ultrasonic scaler to remove calculus and any remaining debris and preserved in a 0.1% thymol solution at 4 °C for a maximum duration of one month prior to testing. Subsequently, root surfaces were enveloped with a 0.2 mm layer of resin gum (Anti-Rutsch-Lack; Wen-ko-Wenselaar GmbH & Co. KG, Hilden, Germany) to replicate the periodontal ligament. Thereafter, they were encased in epoxy resin (Buehler, Lake Bluff, IL, USA) using a rubber mold, aligned along their longitudinal axis, 2.0 mm below the cement-enamel junction (CEJ), facilitated by a dental surveyor (Paraflex Paralelometer; BEGO GmbH & Co. KG, Bremen, Germany).

**Figure 1:**
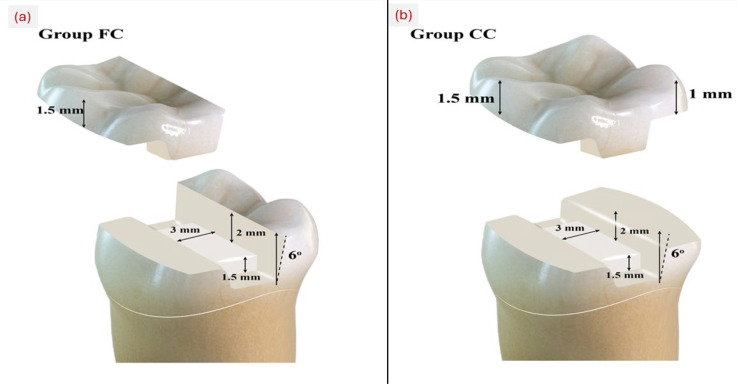
Schematic diagram showing: (**a**) functional cuspal coverage (FC) and (**b**) complete cusp coverage (CC) designs.

A set of diamond burs specifically designed for preparing ceramic onlays (Expert Set 4562; Komet Dental, Gebr. Brasseler GmbH & Co. KG, Lemgo, Germany) was utilized to standardize the tooth preparations. The preparation design followed the manufacturer recommendations for hybrid ceramic onlay restorations current clinical guidelines for occlusal reduction in partial coverage restorations. Specifically, 1.5 mm reduction was applied on functional cusps and 1.0 mm on non-functional cusps to ensure adequate material thickness for load-bearing stability while maintaining conservative tooth reduction. These dimensions are supported by Hofsteenge et al. [5], who demonstrated optimal biomechanical behavior and stress distribution for hybrid ceramics at similar reduction depths. Replacement (refill) burs were utilized after every five preparations to ensure consistent cutting efficiency. Cavities were constructed with the following dimensions: the gingival seat measured 1.5 mm below the pulpal floor, the isthmus width was 3 mm, the central groove depth was 2 mm, and the axial wall taper was set at 6 degrees. Following the standard MOD (Mesio-Occluso-Distal) preparation, a buccal functional cusp reduction of 1.5 mm was executed on 40 mandibular teeth (FC groups) (Figure 1). In contrast, the other 40 mandibular teeth experienced reductions of 1.5 mm on the buccal cusps and 1 mm on the lingual cusps (CC groups) [27] (Figure 1). The occlusal preparation applied to the samples was standardized during preparation using polyvinyl siloxane indices (Express; 3M ESPE, St. Paul, MN, USA). The software preparation check feature was utilized for achieving preparation design purposes. This feature includes tools for analyzing wall smoothness, restoration thickness, and identifying any undercuts or sharp angles. All preparations were thoroughly examined.

After following the basic MOD (Mesio-Occluso-Distal) preparation, buccal functional cusp reduction of 1.5 mm of 40 mandibular teeth was performed (FC groups) (Figure 1), while the remaining 40 mandibular teeth underwent buccal and lingual cusps reduction by 1.5 mm and 1 mm (CC groups) [27] (Figure 1). During preparation, the extent of occlusal preparation applied to the samples was standardized using polyvinyl siloxane indices (Express; 3M ESPE, St. Paul, MN, USA). While as for design, using the software’s preparation check feature, which includes tools for analyzing wall smoothness, restoration thickness, and the identification of any undercuts or sharp or point angles, all preparations were examined.

### 2.3. Adhesive Protocol

All bonding procedures were conducted by a single calibrated operator to eliminate inter-operator variability. The calibration process verified consistency in adhesive application, air-thinning, and light-curing techniques under the supervision of an experienced faculty member. Calibration was completed when the operator exhibited a reproducible technique across three consecutive specimens that met predefined laboratory criteria. Following preparation, enamel margins were selectively etched using 35% phosphoric acid (Select Hv Etch; BISCO Inc., Schaumburg, IL, USA) for 20 s, subsequently rinsed, and dried with compressed air. An adhesive layer (All Bond Universal; BISCO Inc., Schaumburg, IL, USA) was applied to the etched enamel and dentin surfaces utilizing a microbrush. The layer underwent air thinning for 20 s, followed by light curing for 30 s with a calibrated LED curing device (Elipar S10; 3M ESPE, Seefeld, Germany) emitting 1200 mW/cm^2^. The light intensity of the curing unit was consistently measured with the 3M Elipar Radiometer (3M ESPE, Seefeld, Germany).

### 2.4. Digital Impression and Fabrication of Restorations

Cerec Digital impressions of the prepared specimens were acquired via CEREC Omnicam scanner (Dentsply Sirona, Bensheim, Germany). The virtual models were processed with CEREC SW 5.2.1 software (Dentsply Sirona, Bensheim, Germany), utilizing the biogeneric individual option and a spacer setting of 100 µm to maintain consistent anatomical morphology. Restorations were subsequently created utilizing the CEREC MCXL milling equipment (Dentsply Sirona, Bensheim, Germany), resulting in a total of 80 restorations (40 composed of EN and 40 from GC). The specimens were categorized into four experimental groups (*n* = 20 each) according to the combination of cusp coverage design and ceramic material: Group 1—FC with CS (FC-CS), Group 2—FC with EN (FC-EN), Group 3—CC with CS (CC-CS), and Group 4—CC with EN (CC-EN) (Table 1).

### 2.5. Cementation Procedures

Prior to final bonding, the prepolymerized adhesive layer was reactivated using air-particle abrasion with an intraoral sandblaster (Airsonic Mini-Sandblaster; Hager & Werken GmbH & Co. KG, Duisburg, Germany) to restore surface microroughness and promote optimal bonding. The internal surfaces of the EN and GC restorations were pretreated according to manufacturer recommendations. Each was etched with 5% hydrofluoric acid gel (Porcelain Etchant; Ivoclar Vivadent, Schaan, Liechtenstein) for 60 s, thoroughly rinsed with water, and gently dried with oil-free compressed air. A silane coupling agent (BISCO Silane; BISCO Inc., Schaumburg, IL, USA) was applied for 60 s, then gently air-dried. A dual-cure resin cement (Duo-Link Universal; BISCO Inc., Schaumburg, IL, USA) was applied to the internal surfaces of the restorations. Each restoration was seated into the cavity using a static load of 29 N to ensure uniform film thickness. Excess cement was removed using a microbrush. An oxygen-inhibiting gel (Oxyguard II; Kuraray Noritake Dental Inc., Tokyo, Japan) was applied around all restoration margins prior to light-curing. Light polymerization was performed for 20 s each on the buccal, lingual, occlusal, mesial, and distal surfaces.

### 2.6. Chewing Simulation

Specimens underwent thermocycling in distilled water utilizing a Thermocycler THE-1100 (SD-Mechatronik GmbH, Feldkirchen-Westerham, Germany). The procedure comprised 20,000 cycles alternating between 5 ± 2 °C and 55 ± 2 °C, with a 60 s rest time in each bath and a 20 s transfer period, effectively simulating thermal stresses. Subsequently, mechanical fatigue loading was administered using a chewing simulator (Chewing Simulator CS4; SD Mechatronik GmbH, Feldkirchen-Westerham, Germany) for 240,000 cycles at a force of 49 N. The thermocycling protocol (20,000 cycles) is generally considered to simulate approximately two years of intraoral thermal aging, whereas 240,000 masticatory cycles equate to roughly one year of in vivo chewing function [35].

### 2.7. Marginal Adaptation Test

**Figure 2:**
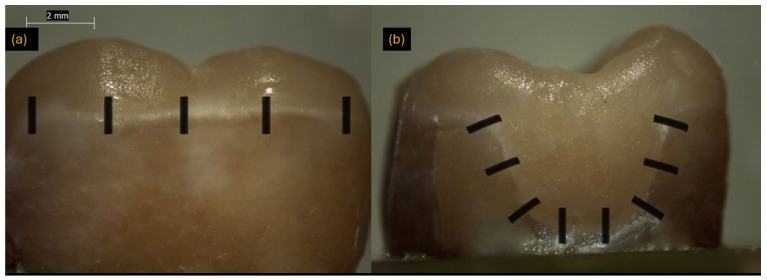
Representative stereomicroscope images used for marginal adaptation measurement. (**a**) Occlusal view showing measurement sites across the occlusal surface (black vertical markers); (**b**) Proximal view showing measurement locations along the marginal interface (black angled markers). Scale bar = 2 mm.

A stereomicroscope (Leica Microsystems; Wetzlar, Germany) at ×10 magnification was used to measure the gap between each tooth and its restoration at 30 predetermined points: eight along the mesial proximal box, eight along the distal proximal box, five along the occlusal-lingual wall, and five along the occlusal-buccal wall (Figure 2). Image analysis was then performed with ImageJ (version 1.40g; Wayne Rasband, NIH, Bethesda, MD, USA) to quantify gap widths accurately.

### 2.8. Fracture Resistance Test

Each specimen was subjected to a compressive load using a universal testing machine (Instron 3366, Norwood, MA, USA) equipped with a 49 N load cell. A stainless-steel rod with a spherical tip with 5.6 mm diameter (radius 2.8 mm) applied force vertically to the center of the restoration at a speed of 1 mm/min until fracture occurred. The maximum load at failure (in Newtons), representing the specimen capacity to withstand compressive force was automatically recorded using Bluehill Lite software (Instron Corp., Norwood, MA, USA).

### 2.9. Failure Mode Analysis

Failure modes were evaluated using a stereomicroscope (Leica Microsystems, Wetzlar, Germany) at ×10 magnification. Failure patterns were independently assessed by two calibrated examiners. Inter-examiner reliability was calculated using Cohen’s kappa (κ = 0.89), indicating strong agreement. Any discrepancies between observers were resolved through discussion and consensus. Restorations were classified into four categories based on established criteria [36]: Type I, restoration fracture with intact tooth; Type II, restoration fracture with minor tooth involvement; Type III, restoration fracture with more than half of the tooth fractured; and Type IV, restoration and tooth fracture involving the root.

### 2.10. Statistical Analysis

Data analysis was performed using IBM SPSS Statistics, version 28. Descriptive statistics were reported as means ± standard deviations for continuous variables and frequencies (percentages) for categorical variables. The normality of data distribution was verified using the Kolmogorov–Smirnov test. Although data normality appeared acceptable, homogeneity of variances (Levene’s test) was not fully met with all variables. In addition, the relatively small sample size per subgroup (*n* = 20) and the presence of slight larger deviations justified the use of nonparametric tests (Kruskal–Wallis followed by Mann–Whitney U tests with Bonferroni correction) to ensure robust comparisons among groups. This approach minimizes Type I error risk under conditions of marginal non-normality or unequal variance. Thus, for comparison of continuous variables (fracture resistance and marginal adaptation) among the four experimental groups (FC-EN, FC-CS, CC-EN, and CC-CS), the Kruskal–Walli’s test was applied. When significant differences were observed, pairwise comparisons were conducted using the Mann–Whitney U test with Bonferroni correction to adjust for multiple testing. A significance level of α = 0.05 was applied to all tests, and all statistical procedures were two-tailed.

## 3. Results

### 3.1. Marginal Adaptation Test

Marginal gap values showed statistically significant differences among the four experimental groups (*p* < 0.001). Pairwise comparisons revealed that restorations fabricated with CS (FC-CS and CC-CS) exhibited significantly larger marginal gaps compared to those fabricated with EN ceramic (FC-EN and CC-EN), irrespective of the preparation design. Specifically, the FC-CS group demonstrated a significantly higher marginal gap compared with FC-EN (*p* < 0.001), and the CC-CS group exhibited a significantly greater gap than CC-EN (*p* < 0.001). However, no significant differences were detected between the same material groups when comparing preparation designs (FC-CS vs. CC-CS, *p* = 1.000); FC-EN vs. CC-EN, *p* = 1.000, 95% confidence intervals) (Table 2).

### 3.2. Fracture Load Measurement (Fracture Resistance Test)

Fracture load (fracture resistance) mean values exhibited statistically significant differences among the four groups (Table 2, (*p* < 0.001)). When categorized by preparation design (Table 3), CC restorations demonstrated significantly higher fracture load compared to FC restorations (*p* < 0.001). When considering both design and material variables (Table 2), CC restorations fabricated using either EN (CC-EN) or GC (CC-CS) hybrid ceramics exhibited significantly higher fracture loads compared to their FC counterparts (FC-EN and FC-CS). No significant differences were observed within each material group with respect to preparation design (FC-EN vs. FC-CS, *p* = 1.000; CC-EN vs. CC-CS, *p* = 0.948). When the results were evaluated based solely on material type (Table 4), no significant difference in fracture load was found between EN and GC hybrid ceramics (*p* = 0.567). There was a significantly lower proportion of Type III & IV failure modes in CS compared to EN (*p*-value = 0.036) (Table 4).

### 3.3. Failure Mode Analysis

**Figure 3:**
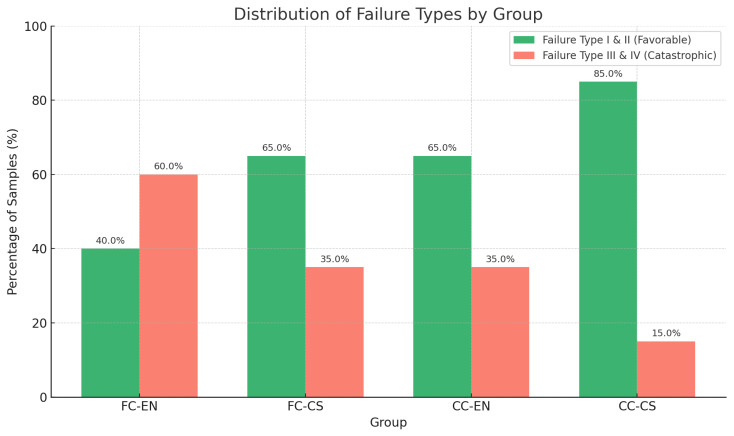
Distribution of failure types (I–IV) across groups. Cusp coverage (CC) groups showed more favorable failures, especially CC-CS (85%), while FC-EN exhibited the highest catastrophic failure rate (60%).

**Figure 4:**
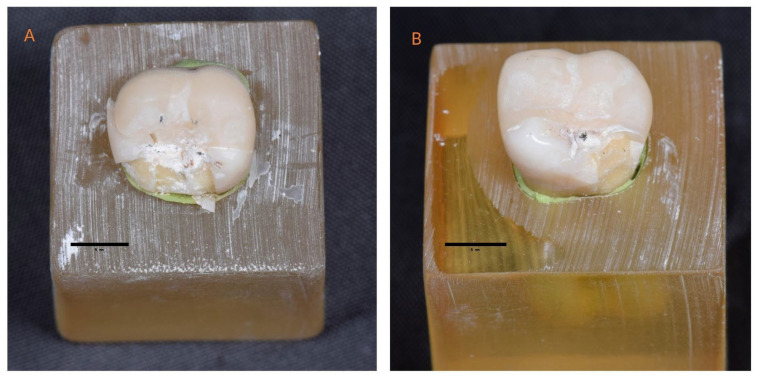
Representative images showing (**A**) Type III failure in a functional cusp coverage restoration fabricated from EN ceramic (FC-EN), characterized by combined restoration and tooth fracture; and (**B**) Type II failure in a functional cusp coverage restoration fabricated from GC Cerasmart (FC-CS), showing cohesive failure limited to the restoration. A unified 5 mm scale bar is included for dimensional reference.

The patterns of failure differed significantly among the study groups (*p* = 0.032). The CC-CS group exhibited no Type IV failures and only two Type I failures, resulting in a combined favorable failure rate (Types I and II) of 85% (95% CI: 62.1–96.8%). In contrast, the FC-EN group demonstrated the lowest proportion of favorable failures (40%, 95% CI: 19.1–63.9%) and the highest incidence of catastrophic failures (Types III and IV), accounting for 60% of specimens (Figure 3 and Figure 4). Within the CC-EN group, two Type IV failures were recorded, while the FC-EN group exhibited one. The distribution of failure types between cusp coverage designs revealed a statistically significant difference (*p* = 0.036), with CC restorations demonstrating a markedly higher proportion of favorable failures compared to FC restorations (75% vs. 52.5%).

### 3.4. Effect Size Analysis

Effect size analysis revealed that cusp coverage design had a very large impact on fracture resistance (Cohen’s d = 3.54), while material type exerted only a small effect (Cohen’s d = 0.31). In contrast, for marginal adaptation, material type had a very large effect (Cohen’s d = 3.05), and cusp coverage showed a small to medium effect (Cohen’s d = 0.42). These findings highlight that biomechanical performance is primarily driven by preparation design, whereas adaptation accuracy is more strongly influenced by the restorative material.

## 4. Discussion

This study evaluated the fracture resistance and marginal adaptation of cusp coverage restorations using two hybrid ceramic materials and two preparation designs after dynamic fatigue loading. The first null hypothesis was rejected, as a statistically significant difference in marginal adaptation was observed between hybrid ceramic materials. Specimens from CS hybrid ceramic displayed greater marginal gaps compared with those from EN ceramic, irrespective of the preparation design. Also, the material composition significantly affects interface adaptation, with a large effect size (Cohen’s d = 3.05). These findings may suggest microstructural differences between EN and CS ceramics to play a role in interface behavior under thermal and mechanical loading.

The lesser values of marginal gaps with EN can be attributed to its feldspathic ceramic network containing aluminum oxide, which enhances dimensional stability, limits hygroscopic expansion, and reduces water sorption [28,29,37]. The higher marginal gaps in CS may be attributed to its resin-rich matrix, which is more susceptible to hydrolytic degradation [33]. The inclusion of monomers such as TEGDMA in hybrid materials may increase water absorption, thereby affecting marginal integrity [20]. Additionally, variations in polymer infiltration levels and filler distribution may contribute to the interfacial gaps. The findings are consistent with previous studies indicating that hybrid ceramics characterized by a higher ceramic fraction and reduced organic content demonstrate enhanced long-term adaptation stability [26,27]. Also, Thermal induced stresses (5–55 °C) may have contributed to this discrepancy [29,37,38]. These findings indicate that ceramics with elevated ceramic-to-polymer ratios may more effectively maintain marginal integrity following the application of aging stresses.

The second null hypothesis was partially accepted, as fracture load values showed statistically significant differences among the experimental groups. In this study, “fracture resistance” if used refers to the maximum fracture load (N) recorded under static compressive testing. While fracture resistance is typically expressed as stress (MPa), the absolute load to fracture was used as a practical indicator of functional strength [3]. Restorations with CC—both with EN and CS—demonstrated significantly higher mean fracture loads compared to their FC counterparts. Although mean fracture loads were comparable between EN and CS, the slightly higher standard deviations observed in the CS suggest greater variability in its mechanical behavior. Furthermore, the mode of failure differed notably between designs. CC restorations exhibited a higher proportion of favorable, repairable fractures, while FC restorations showed more catastrophic root-involving failures.

The large effect size for preparation design (Cohen’s d = 3.54) compared with the small effect for material type (Cohen’s d = 0.31) highlights the biomechanical predominance of design over material in influencing fracture performance. These findings support the view that CC improves failure behavior by absorbing and redirecting occlusal stresses. The findings indicate that CC enhances failure behavior through the absorption and redirection of occlusal stresses. This is consistent with prior research indicating that overlays and endocrowns distribute occlusal loads over a wider surface area, thereby reducing stress concentration in regions subjected to high loads [2,4,13,14,39,40,41]. Kassis et al. [2]. found that complete coverage facilitates cohesive and re-pairable fracture patterns. Thus, CC designs may be appropriate for structurally compromised teeth, including those with significant cuspal loss or those that have undergone endodontic treatment, where managing functional load is essential [42,43,44]. Nonetheless, not all recent research have shown a uniform benefit for CC. Other research indicated that the failure mode primarily dependent on the type of restoration and material selection, which contradicts this study [45,46].

No significant difference in fracture load was observed between EN and CS ceramics when the findings were analyzed exclusively by material type (Table 4). This aligns with the findings of Rexhepi et al. [47] who reported that hybrid ceramics such as EN and CS, due to their polymer-infiltrated microstructures, can absorb stress and resist crack propagation, resulting in comparable failure patterns. However, we disagree with prior findings that indicated a correlation between material selection and fracture mode [45,48]. Other research presented conflicting data for CS hybrid ceramic, which surpassed EN under the imposed loads in one investigation [49]. Some propose that CS exhibits flexural resemblance to dentin and its potential for minimally invasive applications, particularly in instances of worn teeth [50]. The analysis of these data revealed that EN and CS exhibited similar mechanical performance for fracture load, with no impact. Effect size study indicated that material type had a minimal effect (Cohen’s d = 0.31).

Overall, all fracture loads exceeded typical posterior masticatory forces (400–800 N) [17], indicating sufficient strength for clinical application. However, from a clinical perspective, the type of failure may be more relevant than load capacity [3]. In summary, the present findings reinforce that preparation design plays a role in modulating fracture behavior, especially when using brittle ceramics where stress concentration can easily induce catastrophic fracture. Therefore, a synergistic approach combining optimized CC design with a material of compatible modules and strong adhesive integration appears essential to achieving both high fracture resistance and favorable, restorable failure patterns in badly decayed teeth. These observations reinforce the idea that failure mode is not just peak load but should carry high weight in material evaluation. In practice, a repairable fracture (chip, partial break) may preserve the tooth and allow restoration repair, whereas a catastrophic crack or root involvement often mandates extraction or replacement.

This in vitro study has several limitations. Intraoral conditions are not fully replicated by distilled water thermocycling, which lacks the biochemical complexity of saliva. The use of only one dual-cure resin cement limits generalizability. Different resin cements can vary in viscosity, polymerization behavior, and bonding performance, potentially affecting both marginal adaptation and fracture resistance. Future studies should compare multiple adhesive systems to better understand their influence on hybrid ceramic restorations. Baseline marginal adaptation was not recorded pre-aging, making it unclear if discrepancies resulted from thermal/mechanical aging. Moreover, testing was limited to vital mandibular molars; endodontically treated teeth or other tooth types might behave differently under load. Future studies should include clinical trials across diverse scenarios, pre/post-aging assessments, and multiple cementation strategies.

## 5. Conclusions

Based on the results of this in vitro study, the following conclusions were reached:

1. Complete cusp coverage significantly enhanced fracture resistance and resulted in more favorable, repairable failure modes, while functional cusp coverage led to more catastrophic failures. These findings indicate that preparation design plays a more decisive role than material selection in influencing the clinical outcomes of partial-coverage restorations.
2. Both VITA Enamic and GC Cerasmart demonstrated fracture loads exceeding posterior masticatory forces, indicating acceptable clinical strength.
3. VITA Enamic exhibited a higher incidence of catastrophic failures than GC Cerasmart, particularly in restorations with functional cusp coverage.
4. Thermocycling in distilled water may underestimate long-term interfacial degradation, as it does not fully replicate the complexity of the oral environment.

## Figures and Tables

**Table 1:** Description of Hybrid Ceramics Employed in the Study.

Hybrid Ceramic	Composition	Manufacturer
GC Cerasmart	Resin matrix (29% Wt): Bis-MEPP, UDMA, dimethacrylate Inorganic filler (71% Wt): silica, barium glass	GC Corp., Tokyo, Japan
VITA Enamic	Resin matrix (14% Wt): UDMA, TEGDMA Inorganic filler (86% Wt): Feldspathic ceramic network (Al_2_O_3_, Na_2_O, SiO_2_, B_2_O_3_, K_2_O, CaO, ZrO_2_).	Vita-Zahnfabrik, Bad Sackingen, Germany

Abbreviations: Bis-MEPP, Bisphenol A ethoxylate dimethacrylate; UDMA, Diurethane dimethacrylate; TEGDMA, Triethylene glycol dimethacrylate; %Wt means by weight.

**Table 2:** Descriptive statistics of fracture load (N), margin adaptation (µm) and comparison between different fracture patterns in the different groups.

Type of Test	FC-EN (*n* = 20)	FC-CS (*n* = 20)	CC-EN (*n* = 20)	CC-CS (*n* = 20)	*p*-Value
Fracture Load [N]	1699.6 ± 80.2 ^a^	1671.3 ± 53.6 ^a^	1922 ± 91.5 ^b^	1998.5 ± 82.3 ^b^	(<0.001 *)
Margin adaptation [µm]	64.8 ± 4.4 ^a^	80.7 ± 5.1 ^b^	62.7 ± 6.1 ^a^	78.4 ± 5.1 ^b^	(<0.001 *)
Failure Types					
Failure Type I & II	8 (40%)	13 (65%)	13 (65%)	17 (85%)	(0.032)
Failure Type III & IV	12 (60%)	7 (35%)	7 (35%)	3 (15%)	

Different superscript letters (^a^, ^b^) indicate statistically significant differences between groups (*p* ≤ 0.05). *: Significant at *p* ≤ 0.05.

**Table 3:** Descriptive statistics of fracture load (N), margin adaptation (µm) and comparison between different fracture patterns with respect to cusp coverage approach.

Type of Test	FC (*n* = 40)	CC (*n* = 40)	*p*-Value
Fracture load (N)	1685.4 ± 68.8	1960.3 ± 94.2	<0.001 *
Margin adaptation [µm]	72.7 ± 9.3	70.5 ± 9.7	0.363
Failure Types			
Type I &II	21 (52.5%)	30 (75%)	0.036
Type III&IV	19 (47.5%)	10 (25%)	

*: Significant at (*p* ≤ 0.05).

**Table 4:** Descriptive statistics of fracture load (N), margin adaptation (µm) and comparison between different fracture patterns with respect to type of hybrid ceramic.

Type of Test	EN (*n* = 40)	CS (*n* = 40)	*p*-Value
Fracture load (N)	1810.8 ± 141.0	1834.9 ± 179.3	0.567
Margin adaptation [µm]	63.8 ± 5.4	79.5 ± 5.2	<0.001 *
Failure Types			
Type I & II	21 (52.5%)	30 (75%)	0.036
Type III & IV	19 (47.5%)	10 (25%)	

*: Significant at (*p* ≤ 0.05).

## Data Availability

The original contributions presented in this study are included in the article. Further inquiries can be directed to the corresponding authors.

## Abbreviations

The following abbreviations are used in this manuscript:

CAD/CAM	Computer-Aided Design/Computer-Aided Manufacturing
CC	Complete Cusp Coverage
CEJ	Cementoenamel Junction
EN	VITA Enamic (Hybrid Resin–Ceramic)
FC	Functional Cusp Coverage
GC	GC Cerasmart (Hybrid Resin–Ceramic)
HF	Hydrofluoric Acid
LED	Light-Emitting Diode
MOD	Mesio-Occluso-Distal
Mpa	Megapascal
PICN	Polymer-Infiltrated Ceramic Network
